# Identification of tissue-specific cis-regulatory modules based on interactions between transcription factors

**DOI:** 10.1186/1471-2105-8-437

**Published:** 2007-11-09

**Authors:** Xueping Yu, Jimmy Lin, Donald J Zack, Jiang Qian

**Affiliations:** 1 Wilmer Institute, Johns Hopkins University School of Medicine, Baltimore, MD 21287, USA; 2 Department of Molecular Biology and Genetics, Johns Hopkins University School of Medicine, Baltimore, MD 21287, USA; 3 Department of Neuroscience, Johns Hopkins University School of Medicine, Baltimore, MD 21287, USA; 4 McKusick-Nathans Institute of Genetic Medicine, Johns Hopkins University School of Medicine, Baltimore, MD 21287, USA

## Abstract

**Background:**

Evolutionary conservation has been used successfully to help identify cis-acting DNA regions that are important in regulating tissue-specific gene expression. Motivated by increasing evidence that some DNA regulatory regions are not evolutionary conserved, we have developed an approach for cis-regulatory region identification that does not rely upon evolutionary sequence conservation.

**Results:**

The conservation-independent approach is based on an empirical potential energy between interacting transcription factors (TFs). In this analysis, the potential energy is defined as a function of the number of TF interactions in a genomic region and the strength of the interactions. By identifying sets of interacting TFs, the analysis locates regions enriched with the binding sites of these interacting TFs. We applied this approach to 30 human tissues and identified 6232 putative cis-regulatory modules (CRMs) regulating 2130 tissue-specific genes. Interestingly, some genes appear to be regulated by different CRMs in different tissues. Known regulatory regions are highly enriched in our predicted CRMs. In addition, DNase I hypersensitive sites, which tend to be associated with active regulatory regions, significantly overlap with the predicted CRMs, but not with more conserved regions. We also find that conserved and non-conserved CRMs regulate distinct gene groups. Conserved CRMs control more essential genes and genes involved in fundamental cellular activities such as transcription. In contrast, non-conserved CRMs, in general, regulate more non-essential genes, such as genes related to neural activity.

**Conclusion:**

These results demonstrate that identifying relevant sets of binding motifs can help in the mapping of DNA regulatory regions, and suggest that non-conserved CRMs play an important role in gene regulation.

## Background

Transcriptional regulation is a key component of gene regulation, which itself plays a major role in all forms of cellular differentiation and function. To understand the mechanisms that regulate gene expression, it is important to identify and define the network of cis-acting DNA regulatory elements, which can be viewed as the regulatory code wired within the genome. The code itself is executed through transcription factors (TFs), which determine which set of genes will be expressed. Because the cis-regulatory elements are usually short and degenerate, distinguishing regions of regulatory importance from other non-coding regions is often a great challenge.

One important method for cis-regulatory element detection is based on the concept of the cis-regulatory module (CRM). The hypothesis behind this approach is that TFs co-operate as a functional complex in regulating gene expression. A corollary of this hypothesis is that a region with multiple putative TF binding sites (TFBSs) is more likely to be functional than a region with only a solitary site. These studies were carried out by counting TFBS hits in sliding windows along genomic sequence, and then predicting that the regions with the highest density of TFBSs represent CRMs. This CRM concept has been applied to several biological systems [1-9]. Methods based on Hidden Markov Models have been developed to use this CRM concept to improve motif identification [10,11]. A limit that has prevented this approach from being applied on a large scale is the a priori requirement of a set of biologically relevant TFs. Definition of the set of relevant TFs generally requires extensive experimental work, and for most cases such data is not available.

Evolutionary conservation (phylogenetic footprinting approach) can help to improve the cis-regulatory elements identification when used in combination with other methods. This approach is based on the hypothesis that evolutionarily conserved sequences within non-coding regions are the result of selective pressure, and are likely to be enriched for functional regulatory elements. This type of approach has been successfully applied to a variety of systems [12-18]. However, by the nature of the approach, it is not effective for the discovery of species-specific regulatory elements. This limitation is becoming increasingly important because a growing body of evidence suggests the biological importance of non-conserved sequences. Recent comparative genomics studies have identified important RET elements in human and zebrafish that are not conserved evolutionarily [19]. On a genome-wide scale, comparisons between *Drosophila *species show only slight difference in conservation between known regulatory regions and other non-coding regions, again suggesting the regulatory importance of non-conserved sequences [20]. A large scale analysis from the ENCODE project found that only 55% of regulatory factor binding sites overlap the high-confidence evolutionarily constrained sequences, suggesting the possibility of large number of neutral regulatory elements which are biologically functional but are not under selective pressure [21].

To study tissue-specific gene regulation, we have been working to develop approaches to identify and analyze regulatory regions that contribute to tissue specificity. We suggest that species differences in regulatory regions may be particularly important in specialized functions such as the determination of tissue specificity. Therefore, it is important to identify tissue-specific cis-regulatory elements in an unbiased way in terms of evolutionary conservation. In the work described here, the goals were to (1) develop a computational method for identification of cis-regulatory elements that works with both conserved and non-conserved regions, (2) discover regions that are responsible for tissue specificity, and (3) functionally compare conserved and non-conserved regions with the hope of providing a glimpse into the evolution of gene regulation.

Our algorithm, called CRM-PI (cis-regulatory module identification based on protein interaction), searches for DNA regions with dense TFBSs whose binding TFs are known or predicted to be important to tissue specificity. The approach is similar to the above-described methods for mapping CRMs in genomes [1-9], but it has been adapted to deal with situations in which experimental data defining biologically relevant sets of interacting factors is lacking. Sets of putative interacting TFs are first computationally predicted based on the positional relationship and co-occurrences of their binding sites in the conserved regions of the promoters of tissue-specific genes [22]. Based upon these predictions on the trans-acting factors, efforts are made to construct cis-acting DNA regions (i.e. CRMs) in the promoter of each tissue-specific gene, including conserved and non-conserved regions. Since our predicted CRMs do not rely on evolutionary conservation, we are able to investigate the contribution of both conserved and non-conserved CRMs to tissue specific gene regulation, and to study the associated characteristics in the context of the evolution of gene regulation.

## Results

### Detection of CRMs based on tissue-specific TF interactions

**Figure 1:**
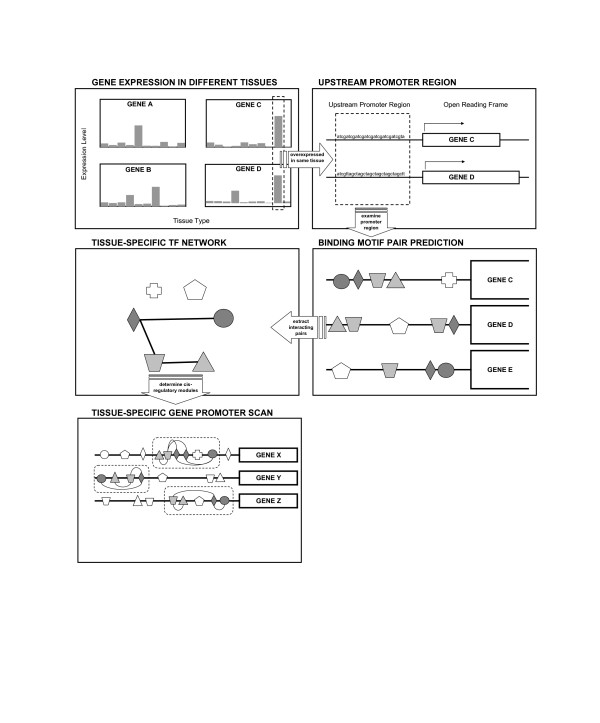
Schematic of module detection method based on TF interactions. Based on gene expression profiles across different tissues, we identified groups of genes that are preferentially expressed in tissues (e.g. gene C and D in the schematic). For each group of genes, we searched the binding sites of known TFs in promoter regions and determined the TF pairs whose binding sites tend to co-occur in close proximity. A tissue-specific TF interaction network was obtained from the analysis. We then scanned the genomic regions and identified cis-regulatory regions (CRMs). The CRMs are defined as regions enriched with TF interactions. Note the first steps were implemented in our previous work [22] while this paper focuses on the last step.

We utilized computationally predicted tissue-specific TF interactions to identify CRMs. In our previous work we identified 9060 putative tissue-specific TF interactions [22]. Two TFs were predicted as interacting if the relative positions and co-occurrence of their binding sites in promoters differed significantly from random expectation (Figure 1; Additional files 1 and 2). Since identifying the CRMs harboring these interactions in each individual promoter is not trivial, we developed an algorithm, CRM-PI, to detect CRMs by calculating an empirical "potential energy" between interacting TFBSs along the genomic sequence. A promoter region containing many interacting TFBSs will have low "potential energy" (see Methods). CRM-PI obtains an energy landscape along the regulatory regions and searches for regions with low "potential energy". For those locations at which the energy is below a given threshold, the region around the minimum is defined as a CRM.

**Figure 2:**
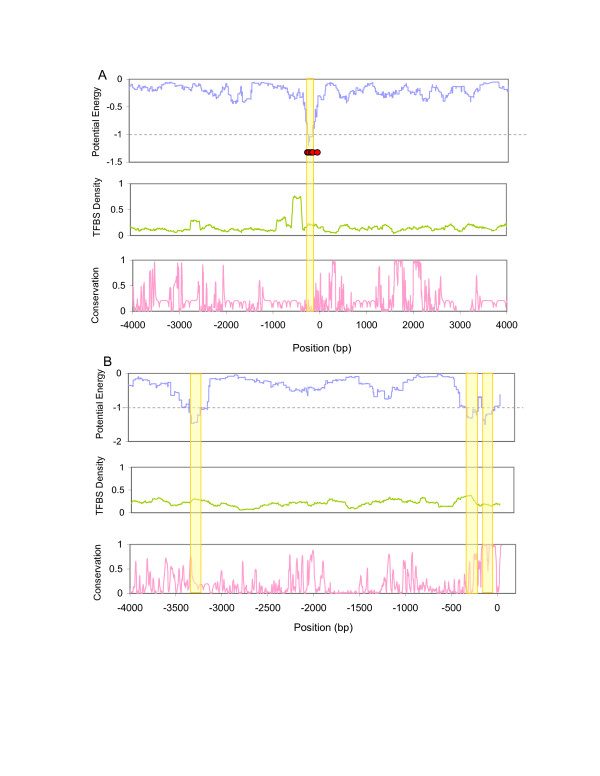
Two examples of predicted CRMs. (A) upstream 5 k to translational start site for gene *ALDOA*. (B) same for gene *CNGB3*. Upper panels are the "potential energy" based on TF interactions. Middle panels show the density of all known TFBSs (total 306 TFBSs) in a sliding window along the region. Bottom panels depict the conservation scores of the regions. The dashed lines are the thresholds used in our prediction. The positions with lower energy than the threshold are predicted as CRMs (indicated by vertical bars). The red dots in (A) indicate the positions of known regulatory sites.

As one example of the use of CRM-PI, Figure 2A shows the promoter region around the gene Aldolase A (*ALDOA*, NM_000034), which is found to be preferentially expressed in the larynx. The energy landscape (*E*_*crm*_) based on larynx-specific TF interactions is shown in the bottom panel of the figure. An energy minimum around -200 bp is lower than the threshold (dashed line) and thus predicts the presence of a CRM (highlighted by vertical bar). Five known cis-regulatory elements are also located around this position (red dots). Interestingly, the region is not evolutionarily conserved (upper panel). Furthermore, if we simply count hits of all TFBSs instead of only the relevant (interacting) ones, the TFBS density at this region is just around the average value.

CRM-PI detected novel as well as known CRMs. For example, we identified three putative CRMs for cyclic nucleotide gated channel beta 3 (*CNGB3*, NM_019098), a gene that is preferentially expressed in the retina. Two CRMs are within upstream 400 bp, while the other one is located around upstream 3200 bp (vertical bars in Figure 2B). The CRM closest to the transcription start site (TSS, 0 on the x-axis) is conserved (average conservation score: 0.92). However, the TFBS density is not high around this region. For the CRM spanning from -200 bp to -340 bp, the region is also relatively conserved. The one at -3200 bp, however, is not conserved, and there is only a minimal increase in TFBS density in this region. These examples demonstrate that our approach can identify CRMs in both conserved and non-conserved regions.

We applied our method to 7261 genes that are preferentially expressed in various human tissues [22]. For each of the 7261 tissue-specific genes, we calculated the energy landscape based on the TF interactions specific to the respective tissues. The investigated regions included sequences upstream of the translation start sites, introns, and sequences downstream of the transcription end sites (see Methods). Among the tissue-specific genes, 2130 of them were found to contain a total of 6232 CRMs, with an average length of 90 bp. The summary statistics for each tissue is shown in Table 1. Detailed information of the predictions can be found in Additional file 3.

**Table 1:** Summary statistics of the predictions for the 30 tissues examined.

**Tissue**	**No. of CRMs**	**No. of target genes**	**Important TFs**
Bladder	58	35	SREBP-1, NRF-1, NF-Y, ETF, MAX
Blood	319	150	FOXJ2, ELF-1, ETF, CDP, PEA3
bone	4	4	OCT-1, RFX1, EF-C, FOXJ2, NKX3A
bone marrow	138	73	SREBP-1, NRF-1, STAT1, HLF, TEF
brain	757	149	FREAC-7, OCT-1, SOX-9, FREAC-3, NKX6-2
cervix	174	90	ETF, NRF-1, SP1, AP-2, C-MYC/MAX
colon	225	68	CDP, AFP1, HNF-1, OCT-1, ALX-4
eye	242	78	FOXJ2, POU3F2, OCT-1, CHX10, CRX
heart	192	62	MEF-2, POU3F2, GATA-6, AP-1, IRF1
kidney	180	83	HNF-1, COUP-TF/HNF-4, CRX, OCT-1
larynx	225	99	ETF, SP1, NRF-1, AP-2, WHN
liver	300	110	HNF-1, ALX-4, HNF-3alpha, C/EBPgamma
lung	64	28	ETF, MTF-1, C-MYC/MAX, LHX3, NRF-1
lymph node	194	111	ICSBP, PU.1, ETF, ELK-1, NRF-1
mammary gland	180	69	RSRFC4, CDP, MEF-2, FAC1, LHX3
muscle	169	69	MEF-2, RSRFC4, SRF, MYOD, CDP_CR3
ovary	121	47	VDR, MAZ, MZF1, SP1, CREB
pancreas	110	48	MYOD, ATF, SP1, E47, AREB6
PNS	260	55	POU3F2, OCT-1, NKX6-2, HNF-6, HFH-3
placenta	121	57	LHX3, AFP1, CHX10, NKX6-2, CDP
prostate	212	64	LHX3, POU3F2, CDC5, C/EBPgamma, CART-1
skin	50	28	AREB6, LMO2_COMPLEX, ALX-4, ARP-1
small intestine	435	68	POU3F2, LHX3, NKX6-2, HNF-1, FOXD3
soft tissue	217	70	FOXO4, C/EBPgamma, FOXO1, SRY, RSRFC4
spleen	94	44	RSRFC4, LBP-1, CDP, MEF-2, NF-KAPPAB
stomach	131	76	ETF, SP1, AP-2gamma, AREB6, SRY
testis	579	296	NRF-1, ETF, SP1, AP-2, C-MYC/MAX
thymus	155	47	POU3F2, NKX6-2, E4BP4, TAX/CREB, ETF
tongue	183	104	ETF, SP1, NRF-1, HIF-1, CREB
uterus	143	28	POU3F2, OCT-1, E4BP4, POU1F1, NKX6-1

**Total**	**6232**	**2310**	

Important TFs of a tissue are the top 5 TFs which contribute most to the potential energies of the CRMs.

### Evaluation using known regulatory elements and DNase I hypersensitive sites

In an effort to evaluate CRM-PI, we first explored its behavior with known cis-regulatory elements. We collected the elements from the TRANSFAC database [23], and chose those in the investigated regions of the tissue-specific genes as the positive controls. In total, we identified 548 regions as positive controls.

Sensitivity and specificity are widely used criteria in evaluation of a prediction. Here, because only a very small fraction of cis-regulatory elements are determined, we calculated enrichment instead of specificity. Sensitivity is defined as the ratio of the recovered positive control regions (in unit of nucleotides) to total length of the positive control regions. The enrichment is defined as the ratio of the probabilities of predicting a nucleotide site in positive control region as part of CRM versus a site in random sequence as CRM. If a method does not have any prediction power, the resulted prediction will have enrichment close to 1.

**Figure 3:**
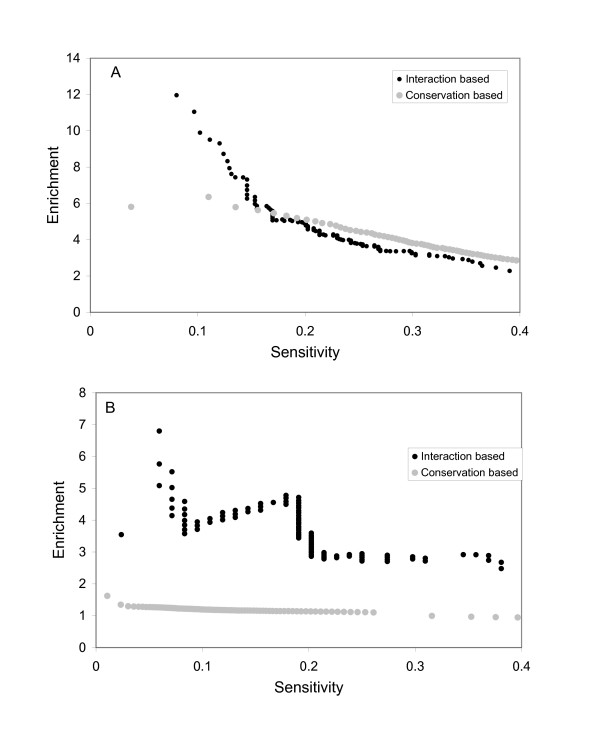
Enrichment and sensitivity of predictions. We evaluated the performance of predictions using sensitivity and enrichment. Two types of predictions were compared: one is the TF interaction based method and the other is the solely conservation based method. (A) Using known regulatory elements as positive controls. (B) Using DNase I hypersensitive sites as positive controls.

We obtained a series of predictions using different thresholds to assess the performance of our approach in terms of sensitivity and enrichment. As expected, if more stringent cutoffs are used, the number of known regulatory regions recovered decreases (Figure 3). At the same time, the enrichment of known regulatory regions in the prediction increases with more stringent cutoffs, indicating that the known regions are not randomly distributed in the prediction set and tend to have more significant "potential energy" from our prediction. This result justifies the usage of "potential energy" as a measure of the relative probability that a region is in a CRM. In our prediction, we chose a threshold of *E*_*crm *_= 1 (see Methods). At this threshold, we have sensitivity of 12% and enrichment of 10. In other words, these CRMs constitute only 1.2% of the regulatory sequences, while 12% of known regulatory regions are found in these CRMs.

To obtain a relative sense of the performance of our approach, we compared our results with an approach that predicts CRMs based purely on conservation. In this simplified comparison, we used conservation scores as the cutoff, and the non-coding regions with conservation scores higher than the cutoff were predicted as CRMs [24]. We found that the performance of our approach was similar to that of this conservation-based method (Figure 3). However, comparison of the approaches revealed that there are significant areas of non-overlap between the sets of identified CRMs, suggesting that the different approaches can complement each other (see Discussion).

We also used DNase I hypersensitive sites (DHSs) as an indirect assessment of our predictions. DHSs represent chromatin regions that show increased sensitivity to digestion by the enzyme DNase I. The increased DNase I sensitivity is thought to reflect areas of DNA where there is increased accessibility to TFs and other DNA binding proteins. DHSs are generally enriched with regulatory elements. We analyzed the 84 DHSs that are associated with tissue-specific genes [25]. Figure 3B shows the sensitivity-enrichment plots for our approach. Our approach can predict DHSs with a reasonable performance. However, an approach based solely on conservation does not effectively distinguish DHSs from the overall genomic sequence (enrichment of DHSs in the prediction is close to 1).

### Some features of predicted CRMs for tissue-specific genes

**Figure 4:**
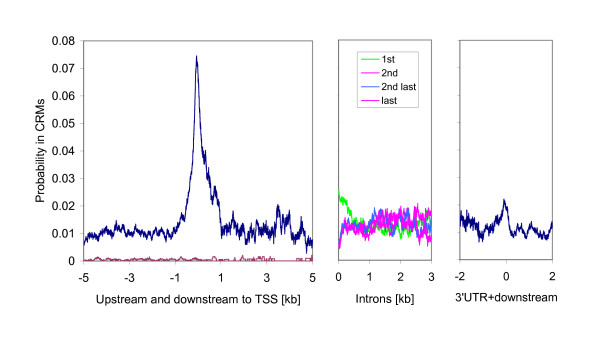
Dependence of regulatory activity on positions relative to gene structure. We calculated the probability for each position containing a CRM. The reference positions (origins in the x-axis) are transcription start sites, the respective start sites of introns and transcription end sites in three regions, respectively. The pink curve in the left panel is from random sequences which were generated with the same nucleic acids compositions and 1^st ^order transition probabilities, respectively, as those of the all promoter sequences in the human genome.

The analysis revealed that CRMs are not homogenously distributed across regulatory regions. We calculated the probability for each position to be in a CRM (Figure 4). There is a significant peak showing high regulatory activity around transcription start sites (TSSs). The peak is located at ~ -60 bp upstream to the TSS. The regulatory activity decays rapidly with increasing distance from the TSS in both directions. The decreases are symmetric for upstream and downstream to TSSs, an observation consistent with the ENCODE analysis [21]. We also noticed that there is a moderate peak at the start position of the first intron. As a comparison, we calculated the same probability for random sequences as the background (pink line in left panel). The random sequences were generated according to the nucleic acid compositions and 1sr/2^nd^/6th order transition probabilities of all promoter sequences in the human genome. The averaged probabilities for a base pair to be in CRM are 0.0005, 0.00056, and 0.0014 for random sequences of orders 1st, 2nd and 6th, respectively. For clarity, only the results of the 1^st^-order random sequences are presented in Figure 4. All regions investigated have significantly higher regulatory activity (>0.01) than the random sequences. This finding is consistent with the results from several large scale ChIP-chip experiments showing that regulatory elements are not restricted to upstream sequences [21,26-28].

We then examined the number of CRMs associated with each gene and found that the CRM numbers display a power-law-like distribution. In other words, most of the genes have only a few associated CRMs and a few genes have many CRMs. More than half of the genes (65%) are associated with only one or two CRMs. On the other hand, only 5 genes (MTTP, MAP2, FSTL5, SLC26A3, and CD36) are regulated by more than 20 CRMs. Of course, these multiple CRMs are not necessary simultaneously active in cells. Different sets of CRMs may be used in different tissues, indicating distinct regulatory mechanisms. For example, CD36 is preferentially expressed in blood, bone marrow, heart, and soft tissue. The numbers of CRMs specific to these tissues are 1, 4, 8, and 9, respectively.

**Figure 5:**
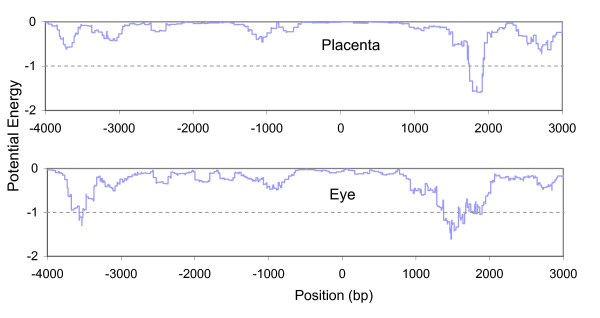
The energy landscapes for PITX2. The landscape in the upper panel was calculated based on placenta-specific interactions between TFs. The one in bottom panel was based on eye-specific TF interactions.

Another example of this phenomenon is provided by *PITX2*, which encodes a paired-like homeodomain transcription factor. *PITX2 *is preferentially expressed in both placenta and eye based on our EST analysis. We calculated the potential energy landscapes using the interaction sets specific to placenta and eye, respectively. Figure 5 shows the energy landscape around the TSS of *PITX2*. The landscape in the upper panel was based on placenta-specific TF interactions. The placenta-specific interactions yield a CRM around 1800 bp in the first intron (the intron spans from 573 bp to 4343 bp). The most important TFs binding to the CRM are LHX3, CHX10 and ATBF1. Similarly, we calculated the energy landscape based on eye-specific interaction for the same gene (bottom panel in Figure 5). Two CRMs were found based on eye-specific TF interactions. One is located around -3600 bp upstream and the other around 1400 bp in the first intron. The TF binding to the CRM in the upstream region is FOXJ2. Three FOXJ2 binding sites occur in the region of the CRM, suggesting several homotypic interactions between FOXJ2. The TFs found in the CRM in intron include FOXJ2, CHX10, POU3F2 and CRX. This example demonstrates that different sets of TFs bind to their respective CRMs and can regulate the same gene in different tissues.

### Conserved and non-conserved CRMs regulate distinct classes of genes

Since our approach to CRM prediction does not rely on evolutionary conservation, one would expect to obtain both conserved and non-conserved CRMs. As expected, the identified CRMs occurred in both conserved and non-conserved regions. Among all predicted CRMs, 55% of them have an average conservation score less than 0.2.

We explored whether there might be differences between the conserved and non-conserved classes of CRMs in terms of the activity and/or properties of their target genes. We first investigated the functional classes enriched for the respective gene groups regulated by conserved CRMs (cCRMs) and non-conserved CRMs (ncCRMs) (see Methods for definitions). We counted the numbers of genes in various functional classes based on gene ontology (GO) annotation [29] and calculated the *P *values for each class (see Methods). Table 2 lists the most significantly enriched functional classes in the gene groups targeted by cCRMs and ncCRMs, respectively. We can see that genes involved in gene expression and protein modification (e.g. transcription, protein amino acid dephosphorylation) tend to be targeted by cCRMs. In contrast, the genes related to nervous system function (e.g. synaptic transmission) tend to be regulated by ncCRMs. The observations on the different enriched functional classes for cCRMs and ncCRMs could be rationalized by the fact that transcription and translation activities are among the most fundamental processes in cells, while neural activity is more specific to species. To exclude possible effects brought about by the conservation of their target genes, we performed the same analysis on the conserved gene groups (of average conservation scores not less than 0.5). One can see that most of the results are consistent with the above (Additional file 4).

**Table 2:** Enriched functional categories in cCRMs and ncCRMs.

	**GO ID**	**GO Description**	**Obs.**	**Exp.**	**-log**_10_**(P)**
**CCRMs**	**GO:0003700**	transcription factor activity	53	26.3	6.0
	**GO:0004840**	ubiquitin conjugating enzyme activity	7	1.3	3.8
	**GO:0006470**	protein amino acid dephosphorylation	12	3.6	3.8
	**GO:0016363**	nuclear matrix	4	0.4	3.7

**NcCRMs**	**GO:0045211**	postsynaptic membrane	12	3.2	5.3
	**GO:0007172**	signal complex formation	6	1.1	4.5
	**GO:0005521**	lamin binding	6	1.1	4.5
	**GO:0007268**	synaptic transmission	31	15.3	4.4

We then linked CRM conservation with the essentiality of their target genes. Essential genes are defined as those that render the cell, or organism, non-viable if knocked out. The essential and viable genes were obtained through mouse knockout experiments [30], and the gene lists themselves were obtained from the Human Protein Reference Database [31]. From the database, we identified 153 viable and 83 essential genes that were tissue-specific and had at least one CRM in their regulatory regions. The fractions of essential genes in the gene group regulated by cCRMs and ncCRMs are 0.48 (45 out 93) and 0.27 (38 out of 143), respectively, suggesting that the classification cCRM/ncCRM is significantly correlated with the classification viable/essential genes (chi square test, *p *< 0.01). Our result demonstrates that the regulatory elements for essential genes tend to evolve slower than those regulating non-essential genes. Since it has been found that more dispensable genes tend to have a higher evolutionary rate [32], this result indicates a possible co-evolution between coding sequences and their regulatory elements.

A recent study on interspecies variation in gene expression revealed that genes containing a TATA box in their promoters tend to have increased gene expression variation across species, indicating that the TATA box could be a genetic signature for gene expression variation [33]. We examined the relationship between the conservation of the CRMs and the likelihood of containing a TATA-box in the promoters. Twelve percent (9 out of 74) of genes regulated by cCRMs contain a TATA-box. In contrast, 23% (39 out of 169) of genes regulated by ncCRMs contain a TATA-box (chi square test, *p *< 0.05). Hypogeometric testing also indicates that the cCRM group significantly overlaps with the non-TATA group (p ~ 0.003) and the ncCRM group with the TATA group (p ~ 0.03). This observation and the above results demonstrate that the genes controlled by cCRMs and ncCMRs are distinctive in terms of functional classification, gene essentiality and the likelihood of being associated with a TATA-box containing promoter.

### Computational complexity and software availability

The computation of a gene's energy landscape takes less than 1 second for a typical 5–10 kbp promoter sequence, and ~42 minutes for ~6000 random sequences in a linux system operating on a DELL PRECISION 690. The program CRM-PI is available upon request.

## Discussion

Gene regulation is not a static process. It is not only about "which TF regulates which genes", but also about "when, where and how the TF regulates the gene". In this context, CRMs are best thought of not just as static segments of DNA sequences located around genes, but rather as dynamic entities that are a function of both time and space. There are many attributes associated with the CRMs, and these attributes are also dynamic. In our analysis, we predicted tissue-specific CRMs, i.e. CRMs associated with gene regulation specific to individual cells or tissues. In different tissues, the CRMs regulating the same gene can be dramatically different (see example in Figure 5). In addition, distinct sets of regulatory elements may regulate the same gene at different developmental stages. As more information becomes available, it should be possible to more fully relate regulatory elements with temporal (e.g. development) and spatial (e.g. cell types) attributes. The additional temporal and spatial information will likely be helpful in describing regulatory elements in their larger biological context.

### Sensitivity

Our approach achieved a reasonable sensitivity of 0.12 for identification of tissue-specific cis-regulatory regions. This performance is similar to other currently available tools. Gupta and Liu tested the CRM predictions of 7 available methods on conserved human-mouse alignment regions of 5 k upstream sequence, and their sensitivities ranged from 0.10 to 0.31 [10]. PreMod is a new promising approach of CRM detection based on conservation, and its sensitivity is 0.15~0.30 for TRANSFAC binding sites.

The modest sensitivity of CRM-PI can be attributed to many factors. First, our approach used 306 TFs with known binding sites, which is just a fraction of the total of ~1500 human TFs [34]. As a result, many CRMs bound by the factors not included this study will be missing from our prediction. Second, our positive controls from TRANSFAC are not necessarily tissue-specific, while our predicted CRMs are only those that contribute to tissue specificity. Third, we expect that if we could include more information for TF interactions, such as binding site orientation and distances, our approach would be more sensitive to detect true CRMs.

### Comparison with other CRM methods

One significant difference between our approach and other CRM methods is that we proposed that only relevant TFBSs contribute to a functional CRM. Therefore, CRM-PI counts the number of interactions instead of the total number of TFBSs in a sliding window for CRM detection. One interesting observation is that we obtained similar densities of TFBSs in promoter sequences and 6^th ^order Markov random sequences (0.152 vs 0.148 hit per bp). In other words, we would have similar numbers of CRMs for real promoters and random sequences if we simply counted the TFBSs. However, the numbers of CRMs (i.e regulatory activities) between the promoter and random sequences are significantly different (> 0.01 vs. 0.0014), suggesting the importance of including only relevant TFs for CRM detection.

### Comparison with conserved-based approach

To enrich for true interactions between TFs, we utilized evolutionary conservation as a constraint [22]. After obtaining the TF interactions, we searched the cis-regulatory regions for each promoter regardless of the degree of evolutionary conservation. As a result, we have found a substantial fraction of CRMs in non-conserved regions, indicating that our approach and conservation-based methods detect different sets of the CRMs. For example, using TFBS regions of TRANSFAC database as positive control, we correctly predicted 66 TFBS regions. Among these 66 sites, only 19 were predicted by PReMod, a genome-wide conservation-based approach [12]. Therefore, our method can complement the widely used conservation-based methods.

### Evolution of regulatory mechanisms

From the perspective of molecular evolution, identification of both conserved and non-conserved regulatory elements can provide insight into the evolution of gene regulation. It has been hypothesized that variation in gene regulation can be as important as coding region changes in contributing to the differences between species [35]. Supporting this viewpoint, human and chimpanzee share 99% sequence identity in coding sequences, yet they still demonstrate major difference in morphology and behavior [36]. Therefore, how genes are regulated might, in some ways, be as critical as evolutionary variation between genes themselves, suggesting that the evolution of regulatory elements might be as important as that of coding sequences in the explanation of divergence among species.

Despite this, there have been few comprehensive studies on the evolution of regulatory elements. In one study, Gasch et al analyzed the conservation and evolution of cis-regulatory systems in fungi [37]. Many cis-regulatory elements in *S. cerevisiae *were found to be conserved in other fungi, but they also found some novel cis-regulatory elements specific to individual species.

One problem limiting comprehensive study of regulatory variation is the lack of sufficient known cis-regulatory elements, especially in mammalian systems. Current studies of the evolution of gene regulation often compare gene expression across species instead of regulatory elements directly [38-40]. It is worth emphasizing that it is not identical to study evolution in terms of gene expression and cis-regulatory elements. For example, a recent study based upon comparison of the messenger RNA levels in liver tissues within and between human, chimpanzee, orangutan and rhesus macaque found that TFs tend to evolve more rapidly than other genes [40]. In contrast, our study on promoter sequences indicates that TFs tend to be regulated by conserved CRMs. Our findings may reflect the evolutionary discrepancy between the gene expression and regulatory elements. The study of regulatory elements therefore requires unbiased (conserved vs. non-conserved) identification of these elements. We suggest that our method will be helpful in the identification of both conserved and non-conserved regulatory elements, which in turn will provide insights into the evolution of regulatory mechanisms.

### Limitations and future directions

In this study, we limited the promoter sequences to 5 kb upstream of the transcription start site (TSS), and thus excluded many enhancers and other regulatory elements that are far from the TSSs. However, extension of our analysis to the entire genomic would likely introduce significant noise, a reflection of the trade-off problem between sensitivity and specificity.

Another area of compromise involved whether or not to include evolutionary conservation. We decided to ignore use of the evolutionary conservation constraint so as to be able to detect both conserved and non-conserved CRMs. A limitation of excluding evolutionary conservation, however, is that we loose its power for helping to identify certain cis-regulatory regions. One possible compromise approach that could take advantage of both approaches would be to include only mammalian genomes to define conservation. In this way, we would have the power of the conservation constraint, and in the meantime, we can hopefully retain most of species-specific cis-regulatory elements.

Our approach to CRM identification can be extended to treat cell-type- or development-stage-specific gene groups. The real challenge of our strategy is how to find well-defined gene groups whose members share similar mechanisms of gene regulation. Secondly, choosing a more non-redundant and comprehensive motif set, such as a refined combination of Jaspar [41] and TRANSFAC, may improve the current performance of our approach. Other developments, such as improvements in motif finding algorithms, may further help improve the global performance of our approach.

## Conclusion

We have presented a study of cis-regulatory systems that regulate tissue-specific gene expression. In contrast to popular phylogenetic footprinting approaches, our proposed method utilizes information based on TF interactions to identify cis-regulatory modules (CRMs), an approach that is unbiased in terms of evolutionary conservation. The results suggest that non-conserved CRMs contribute significantly to tissue-specific gene regulation. With more data available, it should be possible to put the CRMs in a fuller biological context and better understand the roles they play in cellular differentiation and function.

## Methods

### Sequences in the genome

We obtained sequences and gene structures based on RefSeq gene annotation (hg17). The regulatory regions we studied include (i) upstream 5 kb to translation start site (ii) introns (iii) 3'UTR (iv) downstream relative to transcription end site (TES) to its 3' adjacent gene. If the region is longer than 5 kb, we cut it to 5 kb from TES.

### Position weight matrices and genome-wide search

Similar as previous work [22], we collected 306 human position weight matrices (PWMs) from TRANSFAC database and literatures. The match scores between a matrix and a sequence were calculated for all possible positions along the promoters defined in previous work [22]. The score threshold for the top 0.015% matches in all promoters was utilized as the cutoff. This cutoff is somewhat arbitrary and may cause some false positive hits. The study of CRMs based on TF interactions is expected to increase the specificity of these hits.

### Identification of cis-regulatory modules (CRMs)

The basic hypothesis of our method is that the TF complex, rather than individual TFs, is the functional unit of gene regulation. Based on this hypothesis, we perform our analysis by searching for clusters of transcription factor binding sites (TFBSs), which are denoted as cis-regulatory modules (CRMs). These clusters are more likely to be functional than solitary TFBSs. A key step in our method is to identify sets of relevant TFs. We argue that only the clusters of relevant (interacting) TFs are biologically meaningful. Relevant TFs are defined here as the TFs that may interact to co-regulate tissue-specific genes. We identified cis-regulatory modules based on TF interactions. If a region has sufficient TFBSs whose binding TFs interact with each other, we predict the region as CRMs.

In our previous work, we predicted tissue-specific TF interactions [22]. Briefly, we first identified tissue-specific genes based on gene expression profile across various tissues. We then derived TF pairs that are likely to regulate the tissue-specific genes. We scanned known TFBSs (i.e. the binding matrix from TRANSFAC) in the promoters of the tissue-specific genes. If the co-occurrence of two TFBSs is over-represented and/or the distances between the two TFBSs in the promoters are significantly deviating from a random expectation, we predicted the two TFs interact with each other in the tissue (Figure 1). The interaction strength between two motifs is defined as

*S *= -log(*P*) = -log(*P*_*occ*_*P*_*d*_),

where *P*_*occ *_describes whether the co-occurrence (*g*) of the TFBS pair is enriched in the tissue-specific promoters (*n*) compared to the total co-occurrence (*G*) in all *N *promoters.

Pocc=∑x=gG(Gx)(nN)x(1−nN)G−x.
 MathType@MTEF@5@5@+=feaafiart1ev1aaatCvAUfKttLearuWrP9MDH5MBPbIqV92AaeXatLxBI9gBaebbnrfifHhDYfgasaacPC6xNi=xI8qiVKYPFjYdHaVhbbf9v8qqaqFr0xc9vqFj0dXdbba91qpepeI8k8fiI+fsY=rqGqVepae9pg0db9vqaiVgFr0xfr=xfr=xc9adbaqaaeGacaGaaiaabeqaaeqabiWaaaGcbaGaemiuaa1aaSbaaSqaaiabd+gaVjabdogaJjabdogaJbqabaGccqGH9aqpdaaeWbqaamaabmaabaqbaeqabiqaaaqaaiabdEeahbqaaiabdIha4baaaiaawIcacaGLPaaadaqadaqcfayaamaalaaabaGaemOBa4gabaGaemOta4eaaaGccaGLOaGaayzkaaaaleaacqWG4baEcqGH9aqpcqWGNbWzaeaacqWGhbWra0GaeyyeIuoakmaaCaaaleqabaGaemiEaGhaaOWaaeWaaeaacqaIXaqmcqGHsisljuaGdaWcaaqaaiabd6gaUbqaaiabd6eaobaaaOGaayjkaiaawMcaamaaCaaaleqabaGaem4raCKaeyOeI0IaemiEaGhaaOGaeiOla4caaa@4F85@

The contribution from distances between two TFBSs, *P*_*d*_, is obtained from comparison of the observed TFBS-pair distance distribution with that of two random sites using a Kolmogorov-Smirnov (KS) test.

In this work, we then predicted cis-regulatory modules (CRMs) based on the obtained TF interactions. We developed an algorithm, CRM-PI, to calculate an empirical "potential energy" between interacting TFBSs along the genomic sequence. In this context, two TFBSs are considered as interacting if their respective TFs are interacting. Previous methods for identification of CRMs often counted the number of TFBSs in a sliding window along the genomic sequences. Our method is equivalent to count the number of *interactions *in the regions. Note that multiple TFBS matches for the same TF are considered separately. More precisely, we calculated an empirical "potential energy" for each TFBS site,

Ei=−∑jSijexp⁡(−dij/D)
 MathType@MTEF@5@5@+=feaafiart1ev1aaatCvAUfKttLearuWrP9MDH5MBPbIqV92AaeXatLxBI9gBaebbnrfifHhDYfgasaacPC6xNi=xI8qiVKYPFjYdHaVhbbf9v8qqaqFr0xc9vqFj0dXdbba91qpepeI8k8fiI+fsY=rqGqVepae9pg0db9vqaiVgFr0xfr=xfr=xc9adbaqaaeGacaGaaiaabeqaaeqabiWaaaGcbaGaemyrau0aaSbaaSqaaiabdMgaPbqabaGccqGH9aqpcqGHsisldaaeqbqaaiabdofatnaaBaaaleaacqWGPbqAcqWGQbGAaeqaaOGagiyzauMaeiiEaGNaeiiCaaNaeiikaGIaeyOeI0caleaacqWGQbGAaeqaniabggHiLdGccqWGKbazdaWgaaWcbaGaemyAaKMaemOAaOgabeaakiabc+caViabdseaejabcMcaPaaa@4568@

Here *S*_*ij *_is the interaction strength between two TFBSs (see equation 1); *d*_*ij *_is the distance between sites *i *and *j*; *D *is the decaying constant, which is equivalent to the window size from other CRM methods. In our calculations of TF interactions, we found that the average genomic length between two interacting TFBSs is roughly 200 bp, which we choose as the value of *D*. Changing this value in a certain range does affect our results. If the two interacting sites are far away, their contribution to the energy would be small. For each TFBS, we summed up the "potential energy" due to the interactions with all other TFBSs.

In some cases, multiple TFBSs overlap at site *i*, and we choose the TFBS hit whose *E*_*i *_value is the lowest. This *E*_*i *_values still contain the contributions from other overlapped TFBSs. We utilized a simpler approach, compared to previous methods such as AHAB [8] and ClusterDraw [9]. For all sites in the studied sequence, we sort them according to their *E*_*i *_values from low to high, and delete the overlapped hits for each of the ordered sites sequentially. We then update the *E*_*i *_values for the non-overlapped hits. Although this greedy algorithm may not guarantee in obtaining the optimal solution, its efficiency in CPU time makes it a practical choice for a genome-wide calculation.

The *E*_*i *_values for the sites generally fluctuate greatly along the sequence. The lengths of CRMs based on the *E*_*i *_values are often around the length of a TF binding site. To smooth the energy landscape and define a continuous CRM, the contributions from the vicinity of the studied site with a window size *W *were summed up to be as the CRM energy *E*_*crm *_of this site. We chose a window size of *W *= 400 bp. Furthermore, to make the calculations in different tissues comparable, we performed a simulation on random sequences to determine the cutoff for *E*_*crm*_. The random sequences have the same transition probabilities as the sequences (-5000 bp to translation start site) of all RefSeq genes (~23000). For each tissue, we calculated *E*_*crm *_on 10000 random sequences and recorded the lowest *E*_*crm *_value for each random sequence. We chose the cutoff as the *E*_*crm *_which is ranked on 5% lowest values (i.e. the value at the lowest 500) in the random simulation. The *E*_*crm *_was normalized by this value, thus the cutoffs for all tissues are *E*_*crm *_= 1.

### Conservation of sequences and CRMs

We utilized conservation scores, which were calculated based on multiple sequence alignment of eight vertebrate species using the phylogenetic hidden Markov model (phastCons) [42], to evaluate the conservation of sequences and CRMs. The eight species compared were human, chimp, mouse, rat, dog, chicken, fugu, and zebrafish. The conservation score obtained from this comparison reflects the general conservation level within the vertebrates and allows for a metric to determine conservation of larger stretches of genomic sequence. Since CRMs often span several hundred base pairs, the average value of conservation scores of all base pairs in a CRM is an appropriate measurement for the general conservation trend of this CRM.

To determine whether there might be differences between the conserved and non-conserved classes of CRMs in terms of the activity and/or properties of their target genes, we compared the gene groups regulated by conserved CRMs (cCRMs) and non-conserved CRMs (ncCRMs). The cCRM group was defined as those with conservation score > 0.5, and the ncCRM group defined as those with conservation score < 0.2. For those genes controlled by more than one CRM, we used the average conservation score of the associated CRMs. By this definition, 455 and 877 genes were regulated by cCRMs and ncCRMs, respectively. (It should be noted that variation in the cutoffs chosen for the definition of cCRMs and ncCRMs was tested and such variation did not significantly affect the results presented in the result section.)

### Functional enrichment for cCRMs and ncCRMs

We first counted the number of genes associated with cCRMs (or ncCRMs) in each functional class and then compared the observed number with that which would be expected if genes were randomly assigned to the functional class. *P*-values were calculated using the binomial distribution. We did the multiple testing over the functional classes by performing random simulation to determine the cutoff for significant *P*-values. In each simulation we selected a group of genes with the same size of the genes controlled by cCRMs (or ncCRMs) and calculated the *P*-values for their occurrences in different functional classes. We obtained the most significant *P*-value from each simulation and determined the *P*-value at top 5% of the *P*-value distribution from the simulation as the cutoff for significance. For cCRM and ncCRM, the cutoffs for the *P*-values are 10^-3.66 ^and 10^-3.9^, respectively.

## Authors' contributions

JQ and XY conceived of the study. XY implemented the software and performed most of the analysis. JL and DJZ helped to interpret the results. All authors wrote the paper.

## Supplementary Material

Additional file 1 Tissue specific genes. These preferentially expressed genes in 30 human tissues were derived from EST database using methods described in previous work [22]. Click here for file

Additional file 2 Tissue specific TF interactions. These significant TF pairs were calculated according to their overrepresented co-occurrence and distance constraint [22]. Click here for file

Additional file 3 Predictions of tissue specific CRMs. Click here for file

Additional file 4 Enriched functional categories in cCRMs and ncCRMs whose target genes' conservation scores are greater than 0.5. Click here for file

## References

[B1] Berman BP, Nibu Y, Pfeiffer BD, Tomancak P, Celniker SE, Levine M, Rubin GM, Eisen MB (2002). Exploiting transcription factor binding site clustering to identify cis-regulatory modules involved in pattern formation in the Drosophila genome. Proc Natl Acad Sci U S A.

[B2] Berman BP, Pfeiffer BD, Laverty TR, Salzberg SL, Rubin GM, Eisen MB, Celniker SE (2004). Computational identification of developmental enhancers: conservation and function of transcription factor binding-site clusters in Drosophila melanogaster and Drosophila pseudoobscura. Genome Biol.

[B3] Frith MC, Spouge JL, Hansen U, Weng Z (2002). Statistical significance of clusters of motifs represented by position specific scoring matrices in nucleotide sequences. Nucleic Acids Res.

[B4] Halfon MS, Grad Y, Church GM, Michelson AM (2002). Computation-based discovery of related transcriptional regulatory modules and motifs using an experimentally validated combinatorial model. Genome Res.

[B5] Krivan W, Wasserman WW (2001). A predictive model for regulatory sequences directing liver-specific transcription. Genome Res.

[B6] Wasserman WW, Fickett JW (1998). Identification of regulatory regions which confer muscle-specific gene expression. J Mol Biol.

[B7] Zhen Y, Wang Y, Zhang W, Zhou C, Hui R (2007). CardioSignal: a database of transcriptional regulation in cardiac development and hypertrophy. Int J Cardiol.

[B8] Rajewsky N, Vergassola M, Gaul U, Siggia ED (2002). Computational detection of genomic cis-regulatory modules applied to body patterning in the early Drosophila embryo. BMC Bioinformatics.

[B9] Papatsenko D (2007). ClusterDraw web server: a tool to identify and visualize clusters of binding motifs for transcription factors. Bioinformatics.

[B10] Gupta M, Liu JS (2005). De novo cis-regulatory module elicitation for eukaryotic genomes. Proc Natl Acad Sci U S A.

[B11] Zhou Q, Wong WH (2004). CisModule: de novo discovery of cis-regulatory modules by hierarchical mixture modeling. Proc Natl Acad Sci U S A.

[B12] Blanchette M, Bataille AR, Chen X, Poitras C, Laganiere J, Lefebvre C, Deblois G, Giguere V, Ferretti V, Bergeron D, Coulombe B, Robert F (2006). Genome-wide computational prediction of transcriptional regulatory modules reveals new insights into human gene expression. Genome Res.

[B13] Cliften P, Sudarsanam P, Desikan A, Fulton L, Fulton B, Majors J, Waterston R, Cohen BA, Johnston M (2003). Finding functional features in Saccharomyces genomes by phylogenetic footprinting. Science.

[B14] Kellis M, Patterson N, Endrizzi M, Birren B, Lander ES (2003). Sequencing and comparison of yeast species to identify genes and regulatory elements. Nature.

[B15] King DC, Taylor J, Elnitski L, Chiaromonte F, Miller W, Hardison RC (2005). Evaluation of regulatory potential and conservation scores for detecting cis-regulatory modules in aligned mammalian genome sequences. Genome Res.

[B16] Kolbe D, Taylor J, Elnitski L, Eswara P, Li J, Miller W, Hardison R, Chiaromonte F (2004). Regulatory potential scores from genome-wide three-way alignments of human, mouse, and rat. Genome Res.

[B17] Wang T, Stormo GD (2005). Identifying the conserved network of cis-regulatory sites of a eukaryotic genome. Proc Natl Acad Sci U S A.

[B18] Xie X, Lu J, Kulbokas EJ, Golub TR, Mootha V, Lindblad-Toh K, Lander ES, Kellis M (2005). Systematic discovery of regulatory motifs in human promoters and 3' UTRs by comparison of several mammals. Nature.

[B19] Fisher S, Grice EA, Vinton RM, Bessling SL, McCallion AS (2006). Conservation of RET regulatory function from human to zebrafish without sequence similarity. Science.

[B20] Emberly E, Rajewsky N, Siggia ED (2003). Conservation of regulatory elements between two species of Drosophila. BMC Bioinformatics.

[B21] Birney E, Stamatoyannopoulos JA, Dutta A, Guigo R, Gingeras TR, Margulies EH, Weng Z, Snyder M, Dermitzakis ET, Thurman RE, Kuehn MS, Taylor CM, Neph S, Koch CM, Asthana S, Malhotra A, Adzhubei I, Greenbaum JA, Andrews RM, Flicek P, Boyle PJ, Cao H, Carter NP, Clelland GK, Davis S, Day N, Dhami P, Dillon SC, Dorschner MO, Fiegler H, Giresi PG, Goldy J, Hawrylycz M, Haydock A, Humbert R, James KD, Johnson BE, Johnson EM, Frum TT, Rosenzweig ER, Karnani N, Lee K, Lefebvre GC, Navas PA, Neri F, Parker SC, Sabo PJ, Sandstrom R, Shafer A, Vetrie D, Weaver M, Wilcox S, Yu M, Collins FS, Dekker J, Lieb JD, Tullius TD, Crawford GE, Sunyaev S, Noble WS, Dunham I, Denoeud F, Reymond A, Kapranov P, Rozowsky J, Zheng D, Castelo R, Frankish A, Harrow J, Ghosh S, Sandelin A, Hofacker IL, Baertsch R, Keefe D, Dike S, Cheng J, Hirsch HA, Sekinger EA, Lagarde J, Abril JF, Shahab A, Flamm C, Fried C, Hackermuller J, Hertel J, Lindemeyer M, Missal K, Tanzer A, Washietl S, Korbel J, Emanuelsson O, Pedersen JS, Holroyd N, Taylor R, Swarbreck D, Matthews N, Dickson MC, Thomas DJ, Weirauch MT, Gilbert J, Drenkow J, Bell I, Zhao X, Srinivasan KG, Sung WK, Ooi HS, Chiu KP, Foissac S, Alioto T, Brent M, Pachter L, Tress ML, Valencia A, Choo SW, Choo CY, Ucla C, Manzano C, Wyss C, Cheung E, Clark TG, Brown JB, Ganesh M, Patel S, Tammana H, Chrast J, Henrichsen CN, Kai C, Kawai J, Nagalakshmi U, Wu J, Lian Z, Lian J, Newburger P, Zhang X, Bickel P, Mattick JS, Carninci P, Hayashizaki Y, Weissman S, Hubbard T, Myers RM, Rogers J, Stadler PF, Lowe TM, Wei CL, Ruan Y, Struhl K, Gerstein M, Antonarakis SE, Fu Y, Green ED, Karaoz U, Siepel A, Taylor J, Liefer LA, Wetterstrand KA, Good PJ, Feingold EA, Guyer MS, Cooper GM, Asimenos G, Dewey CN, Hou M, Nikolaev S, Montoya-Burgos JI, Loytynoja A, Whelan S, Pardi F, Massingham T, Huang H, Zhang NR, Holmes I, Mullikin JC, Ureta-Vidal A, Paten B, Seringhaus M, Church D, Rosenbloom K, Kent WJ, Stone EA, Batzoglou S, Goldman N, Hardison RC, Haussler D, Miller W, Sidow A, Trinklein ND, Zhang ZD, Barrera L, Stuart R, King DC, Ameur A, Enroth S, Bieda MC, Kim J, Bhinge AA, Jiang N, Liu J, Yao F, Vega VB, Lee CW, Ng P, Shahab A, Yang A, Moqtaderi Z, Zhu Z, Xu X, Squazzo S, Oberley MJ, Inman D, Singer MA, Richmond TA, Munn KJ, Rada-Iglesias A, Wallerman O, Komorowski J, Fowler JC, Couttet P, Bruce AW, Dovey OM, Ellis PD, Langford CF, Nix DA, Euskirchen G, Hartman S, Urban AE, Kraus P, Van Calcar S, Heintzman N, Kim TH, Wang K, Qu C, Hon G, Luna R, Glass CK, Rosenfeld MG, Aldred SF, Cooper SJ, Halees A, Lin JM, Shulha HP, Zhang X, Xu M, Haidar JN, Yu Y, Ruan Y, Iyer VR, Green RD, Wadelius C, Farnham PJ, Ren B, Harte RA, Hinrichs AS, Trumbower H, Clawson H, Hillman-Jackson J, Zweig AS, Smith K, Thakkapallayil A, Barber G, Kuhn RM, Karolchik D, Armengol L, Bird CP, de Bakker PI, Kern AD, Lopez-Bigas N, Martin JD, Stranger BE, Woodroffe A, Davydov E, Dimas A, Eyras E, Hallgrimsdottir IB, Huppert J, Zody MC, Abecasis GR, Estivill X, Bouffard GG, Guan X, Hansen NF, Idol JR, Maduro VV, Maskeri B, McDowell JC, Park M, Thomas PJ, Young AC, Blakesley RW, Muzny DM, Sodergren E, Wheeler DA, Worley KC, Jiang H, Weinstock GM, Gibbs RA, Graves T, Fulton R, Mardis ER, Wilson RK, Clamp M, Cuff J, Gnerre S, Jaffe DB, Chang JL, Lindblad-Toh K, Lander ES, Koriabine M, Nefedov M, Osoegawa K, Yoshinaga Y, Zhu B, de Jong PJ (2007). Identification and analysis of functional elements in 1% of the human genome by the ENCODE pilot project. Nature.

[B22] Yu X, Lin J, Zack DJ, Qian J (2006). Computational analysis of tissue-specific combinatorial gene regulation: predicting interaction between transcription factors in human tissues. Nucleic Acids Res.

[B23] Wingender E, Chen X, Fricke E, Geffers R, Hehl R, Liebich I, Krull M, Matys V, Michael H, Ohnhauser R, Pruss M, Schacherer F, Thiele S, Urbach S (2001). The TRANSFAC system on gene expression regulation. Nucleic Acids Res.

[B24] Pennacchio LA, Rubin EM (2001). Genomic strategies to identify mammalian regulatory sequences. Nat Rev Genet.

[B25] Dorschner MO, Hawrylycz M, Humbert R, Wallace JC, Shafer A, Kawamoto J, Mack J, Hall R, Goldy J, Sabo PJ, Kohli A, Li Q, McArthur M, Stamatoyannopoulos JA (2004). High-throughput localization of functional elements by quantitative chromatin profiling. Nat Methods.

[B26] Cawley S, Bekiranov S, Ng HH, Kapranov P, Sekinger EA, Kampa D, Piccolboni A, Sementchenko V, Cheng J, Williams AJ, Wheeler R, Wong B, Drenkow J, Yamanaka M, Patel S, Brubaker S, Tammana H, Helt G, Struhl K, Gingeras TR (2004). Unbiased mapping of transcription factor binding sites along human chromosomes 21 and 22 points to widespread regulation of noncoding RNAs. Cell.

[B27] Euskirchen G, Royce TE, Bertone P, Martone R, Rinn JL, Nelson FK, Sayward F, Luscombe NM, Miller P, Gerstein M, Weissman S, Snyder M (2004). CREB binds to multiple loci on human chromosome 22. Mol Cell Biol.

[B28] Martone R, Euskirchen G, Bertone P, Hartman S, Royce TE, Luscombe NM, Rinn JL, Nelson FK, Miller P, Gerstein M, Weissman S, Snyder M (2003). Distribution of NF-kappaB-binding sites across human chromosome 22. Proc Natl Acad Sci U S A.

[B29] Ashburner M, Ball CA, Blake JA, Botstein D, Butler H, Cherry JM, Davis AP, Dolinski K, Dwight SS, Eppig JT, Harris MA, Hill DP, Issel-Tarver L, Kasarskis A, Lewis S, Matese JC, Richardson JE, Ringwald M, Rubin GM, Sherlock G (2000). Gene ontology: tool for the unification of biology. The Gene Ontology Consortium. Nat Genet.

[B30] Eppig JT, Bult CJ, Kadin JA, Richardson JE, Blake JA, Anagnostopoulos A, Baldarelli RM, Baya M, Beal JS, Bello SM, Boddy WJ, Bradt DW, Burkart DL, Butler NE, Campbell J, Cassell MA, Corbani LE, Cousins SL, Dahmen DJ, Dene H, Diehl AD, Drabkin HJ, Frazer KS, Frost P, Glass LH, Goldsmith CW, Grant PL, Lennon-Pierce M, Lewis J, Lu I, Maltais LJ, McAndrews-Hill M, McClellan L, Miers DB, Miller LA, Ni L, Ormsby JE, Qi D, Reddy TB, Reed DJ, Richards-Smith B, Shaw DR, Sinclair R, Smith CL, Szauter P, Walker MB, Walton DO, Washburn LL, Witham IT, Zhu Y (2005). The Mouse Genome Database (MGD): from genes to mice--a community resource for mouse biology. Nucleic Acids Res.

[B31] Gandhi TK, Zhong J, Mathivanan S, Karthick L, Chandrika KN, Mohan SS, Sharma S, Pinkert S, Nagaraju S, Periaswamy B, Mishra G, Nandakumar K, Shen B, Deshpande N, Nayak R, Sarker M, Boeke JD, Parmigiani G, Schultz J, Bader JS, Pandey A (2006). Analysis of the human protein interactome and comparison with yeast, worm and fly interaction datasets. Nat Genet.

[B32] Hirsh AE, Fraser HB (2001). Protein dispensability and rate of evolution. Nature.

[B33] Tirosh I, Weinberger A, Carmi M, Barkai N (2006). A genetic signature of interspecies variations in gene expression. Nat Genet.

[B34] Messina DN, Glasscock J, Gish W, Lovett M (2004). An ORFeome-based analysis of human transcription factor genes and the construction of a microarray to interrogate their expression. Genome Res.

[B35] King MC, Wilson AC (1975). Evolution at two levels in humans and chimpanzees. Science.

[B36] Clark AG, Glanowski S, Nielsen R, Thomas PD, Kejariwal A, Todd MA, Tanenbaum DM, Civello D, Lu F, Murphy B, Ferriera S, Wang G, Zheng X, White TJ, Sninsky JJ, Adams MD, Cargill M (2003). Inferring nonneutral evolution from human-chimp-mouse orthologous gene trios. Science.

[B37] Gasch AP, Moses AM, Chiang DY, Fraser HB, Berardini M, Eisen MB (2004). Conservation and evolution of cis-regulatory systems in ascomycete fungi. PLoS Biol.

[B38] Enard W, Khaitovich P, Klose J, Zollner S, Heissig F, Giavalisco P, Nieselt-Struwe K, Muchmore E, Varki A, Ravid R, Doxiadis GM, Bontrop RE, Paabo S (2002). Intra- and interspecific variation in primate gene expression patterns. Science.

[B39] Caceres M, Lachuer J, Zapala MA, Redmond JC, Kudo L, Geschwind DH, Lockhart DJ, Preuss TM, Barlow C (2003). Elevated gene expression levels distinguish human from non-human primate brains. Proc Natl Acad Sci U S A.

[B40] Gilad Y, Oshlack A, Smyth GK, Speed TP, White KP (2006). Expression profiling in primates reveals a rapid evolution of human transcription factors. Nature.

[B41] Sandelin A, Alkema W, Engstrom P, Wasserman WW, Lenhard B (2004). JASPAR: an open-access database for eukaryotic transcription factor binding profiles. Nucleic Acids Res.

[B42] Siepel A, Bejerano G, Pedersen JS, Hinrichs AS, Hou M, Rosenbloom K, Clawson H, Spieth J, Hillier LW, Richards S, Weinstock GM, Wilson RK, Gibbs RA, Kent WJ, Miller W, Haussler D (2005). Evolutionarily conserved elements in vertebrate, insect, worm, and yeast genomes. Genome Res.

